# In vivo assembly and trafficking of olfactory Ionotropic Receptors

**DOI:** 10.1186/s12915-019-0651-7

**Published:** 2019-04-17

**Authors:** Liliane Abuin, Lucia L. Prieto-Godino, Haiyun Pan, Craig Gutierrez, Lan Huang, Rongsheng Jin, Richard Benton

**Affiliations:** 1 Center for Integrative Genomics, Génopode Building, Faculty of Biology and Medicine, University of Lausanne, CH-1015 Lausanne, Switzerland; 2 Department of Physiology and Biophysics, University of California, Irvine, CA 92697 USA; 3 Present address: The Francis Crick Institute, 1 Brill Place, London, NW1 1BF UK; 4 Conagen, 15 DeAngelo Dr, Bedford, MA 01730 USA

## Abstract

**Background:**

Ionotropic receptors (IRs) are a large, divergent subfamily of ionotropic glutamate receptors (iGluRs) that are expressed in diverse peripheral sensory neurons and function in olfaction, taste, hygrosensation and thermosensation. Analogous to the cell biological properties of their synaptic iGluR ancestors, IRs are thought to form heteromeric complexes that localise to the ciliated dendrites of sensory neurons. IR complexes are composed of selectively expressed ‘tuning’ receptors and one of two broadly expressed co-receptors (IR8a or IR25a). While the extracellular ligand-binding domain (LBD) of tuning IRs is likely to define the stimulus specificity of the complex, the role of this domain in co-receptors is unclear.

**Results:**

We identify a sequence in the co-receptor LBD, the ‘co-receptor extra loop’ (CREL), which is conserved across IR8a and IR25a orthologues but not present in either tuning IRs or iGluRs. The CREL contains a single predicted *N*-glycosylation site, which we show bears a sugar modification in recombinantly expressed IR8a. Using the *Drosophila* olfactory system as an in vivo model, we find that a transgenically encoded IR8a mutant in which the CREL cannot be *N*-glycosylated is impaired in localisation to cilia in some, though not all, populations of sensory neurons expressing different tuning IRs. This defect can be complemented by the presence of endogenous wild-type IR8a, indicating that IR complexes contain at least two IR8a subunits and that this post-translational modification is dispensable for protein folding or complex assembly. Analysis of the subcellular distribution of the mutant protein suggests that its absence from sensory cilia is due to a failure in exit from the endoplasmic reticulum. Protein modelling and in vivo analysis of tuning IR and co-receptor subunit interactions by a fluorescent protein fragment complementation assay reveal that the CREL *N*-glycosylation site is likely to be located on the external face of a heterotetrameric IR complex.

**Conclusions:**

Our data reveal an important role for the IR co-receptor LBD in control of intracellular transport, provide novel insights into the stoichiometry and assembly of IR complexes and uncover an unexpected heterogeneity in the trafficking regulation of this sensory receptor family.

**Electronic supplementary material:**

The online version of this article (10.1186/s12915-019-0651-7) contains supplementary material, which is available to authorized users.

## Background

Ionotropic receptors (IRs) are a subfamily of ionotropic glutamate receptors (iGluRs) [1], an ancient class of ligand-gated ion channels present in animals, plants and prokaryotes [2–4]. Comparative genomics analyses suggest that IRs evolved in the last common ancestor of protostomes and probably derived from the AMPA/Kainate clade of iGluRs [5], which have well-characterised roles in synaptic transmission in animal nervous systems [3]. In contrast to these iGluRs, IR repertoires have greatly expanded in size and display high sequence diversity within and between species [5]. Moreover, transcriptomic and in situ investigations in a range of animals indicate that *Ir* genes are expressed in peripheral, rather than central, neurons [5–9]. Functional studies of IRs, in particular in *Drosophila melanogaster*, have shown that these receptors have diverse roles in environmental sensing, including in olfaction [6, 10–12], gustation [13–21], hygrosensation [22–24] and thermosensation [25–27].

**Fig. 1:**
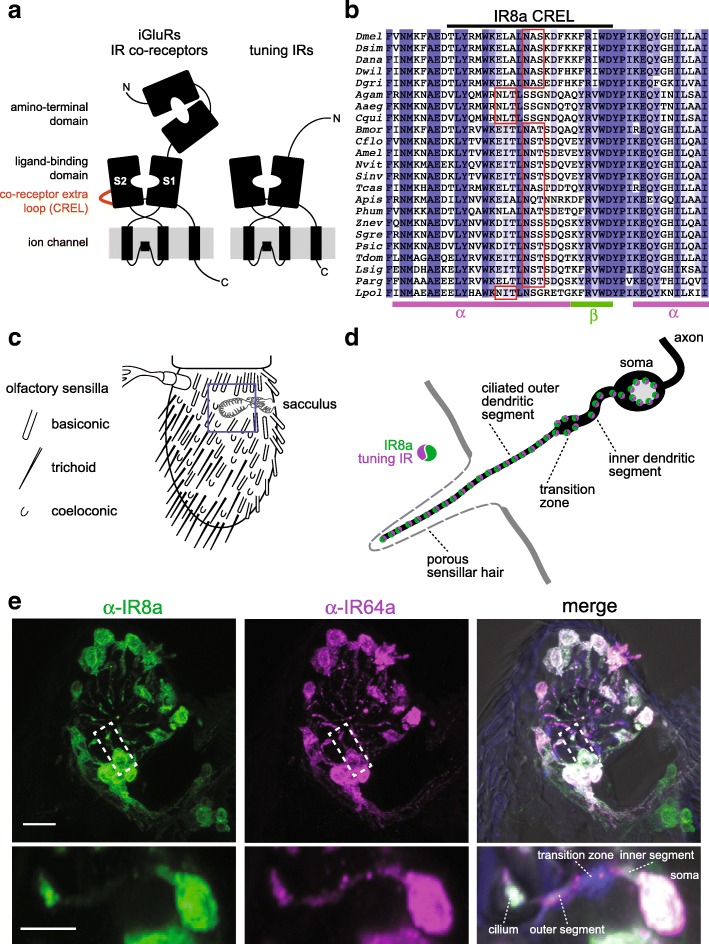
The IR co-receptors contain a distinctive *N*-glycosylated loop. **a** Schematic of the domain organisation of iGluRs, IR co-receptors and tuning IRs. **b** Alignment of the protein sequence spanning the CREL (co-receptor extra loop; black bar) of IR8a orthologues from the indicated species. Predicted *N*-glycosylation sites are highlighted with red boxes and predicted secondary structure is shown below the alignment. Species (top-to-bottom): *Drosophila melanogaster*, *Drosophila simulans*, *Drosophila ananassae*, *Drosophila willistoni*, *Drosophila grimshawi*, *Anopheles gambiae*, *Aedes aegypti*, *Culex quinquefasciatus*, *Bombyx mori*, *Camponotus floridanus*, *Apis mellifera*, *Nasonia vitripennis*, *Solenopsis invicta*, *Tribolium castaneum*, *Acyrthosiphon pisum*, *Pediculus humanus*, *Zootermopsis nevadensis, Schistocerca gregaria*, *Phyllium siccifolium*, *Thermobia domestica*, *Lepismachilis y-signata*, *Panulirus argus*, *Limulus polyphemus*. **c** Schematic of the *Drosophila* third antennal segment showing the distribution of different olfactory sensilla and the internal sacculus. **d** Schematic illustrating the main anatomical features of an olfactory sensory neuron (OSN); the morphology of the cuticular hair and the branched nature of the cilium varies between different sensilla classes (note: most sensilla contain more than one neuron per hair). **e** Immunofluorescence with antibodies against IR8a (green) and IR64a (magenta) on an antennal section of a wild-type animal, showing the region containing the third chamber of the sacculus (blue boxed area in **c**). In the merged image, the transition zone is marked by monoclonal antibody 21A6 (blue), and a bright-field image is overlaid to reveal cuticular anatomical landmarks. Scale bar: 10 μm. The images shown below are of a single OSN (from a subset of optical slices of the area indicated by the dashed white boxes) in which the main anatomical features are shown. Scale bar: 5 μm

Within IR repertoires, two members, IR8a and IR25a, exhibit several distinctive properties: first, they have the highest sequence identity and closest structural organisation to iGluRs [5], comprising an amino-terminal domain (ATD), a ligand-binding domain (LBD) and a transmembrane ion channel domain (Fig. 1a). By contrast, most other IRs lack the ATD and display very low sequence identity to iGluRs, especially within the LBD (Fig. 1a) [1, 5]. Second, these two receptors are deeply conserved, with unambiguous orthologues present across arthropods (for IR8a) or protostomes (for IR25a) [5]. Third, while many IRs are restricted to small populations of sensory neurons, IR8a and IR25a are expressed in multiple, functionally distinct neuron classes [1, 28]. Finally, genetic analysis in *D. melanogaster* indicates that loss of either IR8a or IR25a abolishes the responses of diverse sensory neuron types [16, 19–23, 25, 28, 29]. These observations have led to a model in which IR8a and IR25a function as co-receptors that form heteromeric complexes with distinct sets of selectively expressed, ‘tuning’ IRs, which determine the sensory response specificity of the complex [28].

While some progress has been made in defining the molecular basis of tuning IR response specificity [10, 11], the assembly and trafficking of IR complexes in vivo remain poorly understood. One intriguing unresolved question is the role of the co-receptor LBD. In heteromeric iGluR complexes, each subunit is thought to bind an extracellular ligand (typically glutamate or glycine) and to contribute to the gating of the ion channel pore [2, 3, 30]. It is unlikely that the IR co-receptors bind to the diversity of ligands that activate neurons in which they are expressed (which are presumed to be recognised by the LBD of the particular tuning IR in a given neuron type [10, 11, 28]). One possibility is that the IR co-receptor LBD interacts with a ligand present ubiquitously in the extracellular lymph fluid that bathes the ciliated outer dendritic segment of IR-expressing sensory neurons. The LBDs of both IR8a and IR25a retain most of the principal glutamate-contacting residues of iGluRs [1], raising the possibility that glutamate or a structurally related molecule is such a ligand. However, the observation that several of these residues are dispensable for the function of IR8a [28] implies that the co-receptor LBD has a role that is independent of ligand interactions.

## Results

### IR co-receptor LBDs contain a distinctive *N*-glycosylated protein loop

To investigate the role of the IR co-receptor LBD, we first examined the sequence of this region for any unusual structural features. As in iGluRs, the LBD of IRs consists of a ‘Venus fly trap’-like structure formed by two lobes (S1 and S2), which are separated in the primary sequence by the ion channel domain (Fig. 1a). We generated a multiprotein sequence alignment of the predicted LBD of diverse *D. melanogaster* IRs, including the co-receptors IR8a and IR25a, various tuning IRs and selected mammalian iGluRs. This alignment revealed the presence of a stretch of ~ 30–35 amino acids near the beginning of the S2 domain in IR8a and IR25a that are not aligned to either tuning IR or iGluR sequences (Additional file 1: Figure S1). We termed this region the ‘co-receptor extra loop’ (CREL) (Fig. 1a). The CREL is highly conserved across orthologous co-receptors from diverse species (Fig. 1b and Additional file 2: Figure S2). Consistent with the overall relatedness of the co-receptors, the IR8a and IR25a CRELs share several characteristics, including the presence of predicted short alpha-helical and beta-sheet regions and a single consensus *N*-glycosylation target motif (NXS/T) (Fig. 1b and Additional file 2: Figure S2). Although the *N*-glycosylation site motif is conserved in all CREL sequences in IR8a and IR25a, its position varies by precisely four amino acids in a small subset of orthologues (Fig. 1b and Additional file 2: Figure S2). As this motif is located in a putative alpha-helical region, this displacement would be predicted to maintain the site on the same face of the helix.

We next sought to determine whether the CREL can be *N*-glycosylated. Because of the prohibitively limited quantities of protein we could obtain from tissues in vivo, we used HEK293 cells to express and purify recombinant IR8a LBD (corresponding to the termite *Zootermopsis nevadensis* sequence, which was the most promising candidate of several IR8a orthologues screened). We split the sample in two, treated one with peptide-*N*-glycosidase F (PNGase F), and subjected both to SDS-PAGE with in-gel tryptic digestion before analysis using liquid chromatography-tandem mass spectrometry (LC-MS/MS). When treated with PNGase F, *N*-linked glycans are removed from glycosylated asparagine residues, which become deamidated to aspartic acid (resulting in an increase in peptide mass by 1 mass unit) [31]. Thus, an increase in the abundance of tryptic peptides containing aspartic acid following PNGase F treatment is indicative that these sequences originally contained a glycosylated asparagine. By contrast, such treatment does not affect peptides containing unmodified asparagine residues, whose abundance should therefore remain unchanged. For the IR8a LBD, we identified a tryptic peptide (m/z 648.2984^2+^) whose peak intensity increased ~ 1000-fold after PNGase F treatment (Additional file 3: Figure S3). Subsequent analysis determined its sequence as DITLN*SSSDQSK (where N* refers to a deamidated asparagine residue), which corresponds to the predicted *N*-glycosylation site of the CREL (Additional file 3: Figure S3A and Fig. 1b). An adjacent tryptic peptide (m/z 676.3276^2+^; sequence N*AEDVLYNVWK) had a similar abundance in the untreated and PNGase F-treated samples (Additional file 3: Figure S3B), indicating that this sequence is not *N*-glycosylated. These data indicate that the predicted CREL *N*-glycosylation site can bear a sugar modification.

### The IR8a CREL *N*-glycosylation site has a selective role in regulating subcellular trafficking

To determine whether the CREL and the CREL *N*-glycosylation site are required for IR function in vivo, we focused on *D. melanogaster* IR8a, because the tuning receptor partners of this co-receptor (i.e. acid-sensing IRs in the antenna, the main olfactory organ of insects) are better understood than for IR25a [10, 11, 28, 32]. We generated transgenes encoding *N*-terminally EGFP-tagged mutant versions of IR8a bearing either a small deletion of the CREL (removing T658-D681) or a point mutant that disrupts the *N*-glycosylation site (N669Q), as well as a wild-type IR8a control. These transgenes (*UAS-EGFP:Ir8a*^*wt*^, *UAS-EGFP:Ir8a*^*∆CREL*^ and *UAS-EGFP:Ir8a*^*N669Q*^) were inserted at the same location in the *D. melanogaster* genome to eliminate any positional effects on their expression.

We first expressed these transgenes in olfactory sensory neurons (OSNs) under the control of the *Ir8a*-*Gal4* driver [28]. OSN dendrites are housed within cuticular sensilla that cover the external surface of the antenna as well as lining the sacculus, an internal multichambered pocket (Fig. 1c, d). We focused our attention initially on the subpopulation of IR8a-positive sacculus OSNs that co-express the tuning receptor IR64a [12], because the larger soma and dendrites of these neurons—compared to other IR8a-expressing OSNs that innervate coeloconic sensilla [1]—facilitate observation of subcellular protein distribution (Fig. 1e).

**Fig. 2:**
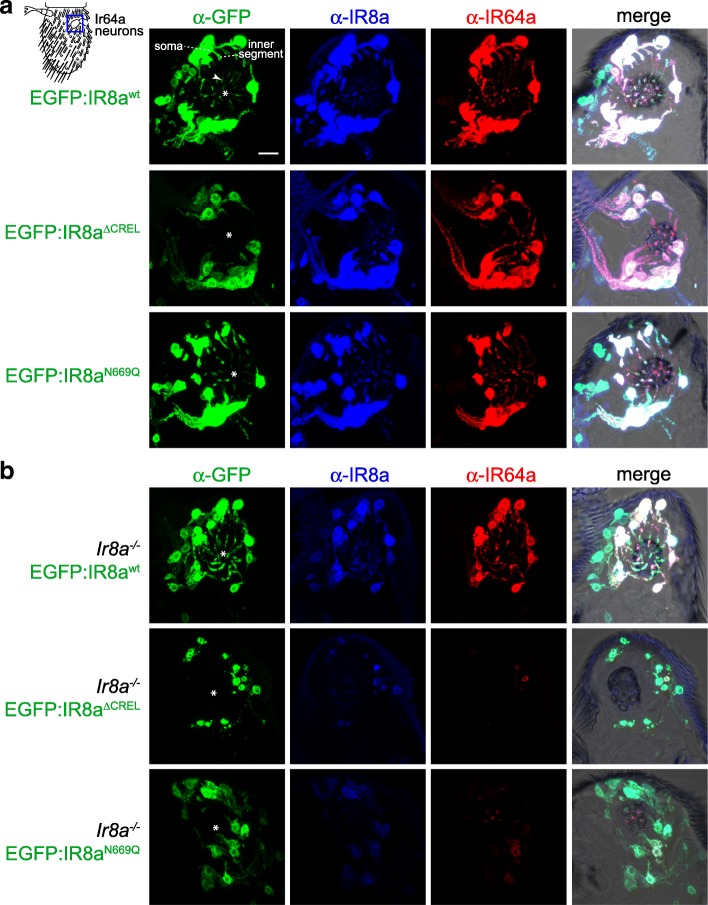
The IR8a CREL functions in subcellular trafficking. **a** Immunofluorescence with antibodies against GFP (green), IR8a (blue) and IR64a (red) on antennal sections of animals expressing the indicated transgenes in Ir8a neurons. Genotypes are of the form Ir8a-Gal4/UAS-EGFP:Ir8a^x^. The white asterisks (in this and other panels) indicate the central cavity of sacculus chamber 3, into which the IR64a+IR8a-expressing OSN ciliated dendrites project (see also the merged panels, in which bright-field images are overlaid to provide anatomical landmarks). In the top left panel, the arrowhead marks the ciliated ending of one neuron; the soma and inner segment of this neuron are also indicated (the outer segment—before the cilium—is difficult to see because only trace levels of receptors are detected in this region). Scale bar (for all panels in this figure): 10 μm. For each genotype, the phenotype was assessed in multiple sections of antennae from at least 20 animals from two independent genetic crosses, allowing observation of several hundred different neurons. We quantified the localisation properties by counting the number of sensory cilia with detectable EGFP signal as a percentage of the total number of cell bodies in the imaged samples; this is not expected to be 100% because sensory endings for each OSN soma are not necessarily present in the thin tissue sections (see ‘Methods’ section on imaging): EGFP:IR8a^wt^ = 75% (83 labelled cilia/111 soma), EGFP:IR8a^∆CREL^ = 0% (0/93), EGFP:IR8a^N669Q^ = 61% (65/106). **b** Immunofluorescence with antibodies against GFP (green), IR8a (blue) and IR64a (red) on antennal sections of animals expressing the indicated transgenes in Ir8a neurons in an Ir8a mutant background. Genotypes are of the form Ir8a^1^/Y;Ir8a-Gal4/UAS-EGFP:Ir8a^x^. EGFP:IR8a^∆CREL^ and EGFP:IR8a^N669Q^are impaired in localisation to the cilia in the absence of endogenous IR8a (the occasional projections from the soma represent protein within the inner segment only). In addition, both proteins appear to be destabilised; consequently, endogenous IR64a is also detected at substantially lower levels in these two genotypes (but see Additional file 4: Figure S4). OSNs that express EGFP:IR8a^∆CREL^ also display signs of sickness (e.g. smaller soma). For each genotype, the phenotype was assessed in multiple sections of antennae from at least 20 animals from two independent genetic crosses. Quantifications: EGFP:IR8a^wt^ = 79% (177/225), EGFP:IR8a^∆CREL^ = 0% (0/198), EGFP:IR8a^N669Q^ = 35% (78/220)

EGFP:IR8a^wt^ displayed a very similar distribution to endogenous IR8a and IR64a in the soma, inner dendritic segment and sensory cilia of the sacculus neurons (Fig. 1e and Fig. 2a) [28]. By contrast, EGFP:IR8a^∆CREL^ was detected in the soma, but never in the sensory cilia where endogenous IR8a and IR64a are found (Fig. 2a; see legend for quantifications). This result indicates a critical role for the CREL in protein folding, complex assembly and/or subcellular localisation. The CREL *N*-glycosylation site mutant, EGFP:IR8a^N669Q^, could however be detected in the sensory compartment, albeit at reduced levels compared to EGFP:IR8a^wt^ (Fig. 2a).

As these neurons also express endogenous IR8a, we next expressed these transgenes in an *Ir8a* mutant background. As observed previously, EGFP:IR8a^wt^ localised normally while EGFP:IR8a^∆CREL^ failed to be targeted to the sensory cilia and was destabilised (Fig. 2b). This protein instability appears to be a secondary consequence of the failure in localisation because examination of neurons in young (< 1 day old) animals did not reveal such a difference in protein levels, despite their absence from cilia (Additional file 4: Figure S4). Unexpectedly, in the absence of wild-type IR8a, EGFP:IR8a^N669Q^ displayed much stronger defects in cilia localisation (Fig. 2b). These observations indicate that the presence of endogenous IR8a can complement the localisation defect of EGFP:IR8a^N669Q^. This result has two implications: first, that within cilia-localised IR complexes, there must be at least two IR8a subunits, providing in vivo evidence for the stoichiometry of IR complexes suggested by in vitro experiments [28]. Second, the ability of EGFP:IR8a^N669Q^to localise to sensory cilia in the presence of IR8a indicates that this mutant protein is not defective in either folding or assembly into transport-competent IR complexes. Rather, the post-translational modification site in the CREL must have a selective effect on subcellular trafficking.

### Heterogeneous requirement for the IR8a CREL *N*-glycosylation site in the localisation of tuning IRs

To examine the role of the IR8a CREL in trafficking of other tuning IRs, we surveyed the localisation of these three EGFP:IR8a fusion proteins in Ir8a neurons across the antenna. While EGFP:IR8a^∆CREL^ was never detected in sensory cilia (in several hundred OSNs visualised), EGFP:IR8a^N669Q^ could be observed in the endings of a subset of neurons innervating coeloconic sensilla, even in the absence of endogenous IR8a (Additional file 5: Figure S5A-B). We reasoned that this differential trafficking ability of IR8a^N669Q^ might be related to the tuning IR with which it is co-expressed.

**Fig. 3:**
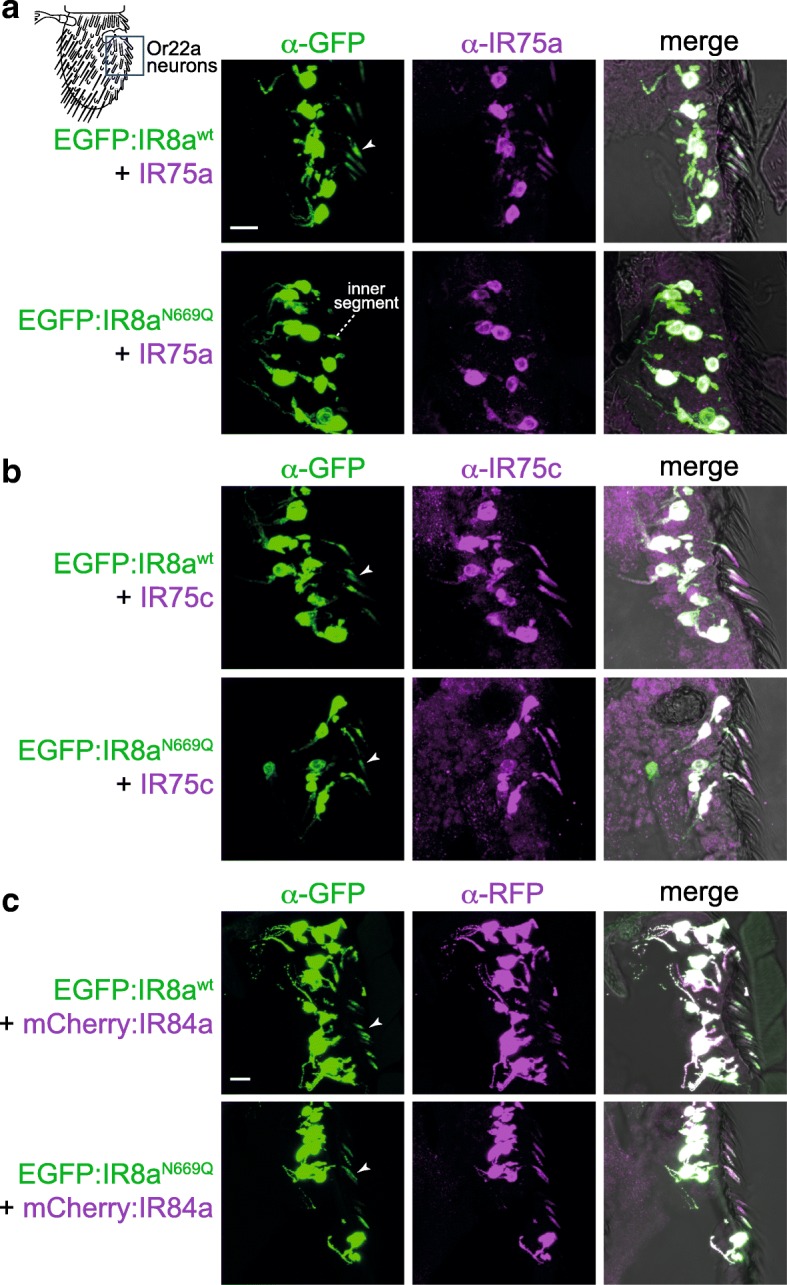
Heterogeneous requirement for the IR8a CREL *N*-glycosylation site in the localisation of tuning IRs. **a** Immunofluorescence with antibodies against GFP (green) and IR75a (magenta) on antennal sections of animals expressing the indicated transgenes in Or22a neurons (representing the field-of-view indicated in the cartoon). The arrowheads (in this and other panels) indicate the cilia of Or22a neurons; in neurons expressing EGFP:IR8a^N669Q^ + IR75a (second row), the receptors are not detected in this sensory compartment, remaining restricted to the inner segment. Receptor localisation was determined by overlaying the fluorescence signal onto a bright-field channel, as shown in the merged images. Note that not all soma have a corresponding ciliated ending in these images, because this is a thin (14 μm) tissue section that does not include the entirety of all neurons. Genotypes are of the form *UAS-EGFP:Ir8a*^*x*^/*UAS-Ir75a;Or22a-Gal4/+*. Scale bar (for panels **a**, **b**): 10 μm. For each genotype, the phenotype was assessed in multiple sections of antennae from at least 20 animals from two independent genetic crosses. **b** Immunofluorescence with antibodies against GFP (green) and IR75c (magenta) on antennal sections of animals expressing the indicated transgenes in Or22a neurons. Genotypes are of the form *UAS-EGFP:Ir8a*^*x*^/*UAS-Ir75c;Or22a-Gal4/+*. For each genotype, the phenotype was assessed in multiple sections of antennae from at least 20 animals from two independent genetic crosses. **c** Immunofluorescence with antibodies against GFP (green) and RFP (magenta) on antennal sections of animals expressing the indicated transgenes in Or22a neurons. Genotypes are of the form *UAS-EGFP:Ir8a*^*x*^*/+;Or22a-Gal4/UAS-mCherry:Ir84a*. Scale bar: 10 μm. For each genotype, the phenotype was assessed in multiple sections of antennae from at least 30 animals from three independent genetic crosses

To test this possibility, we used an in vivo heterologous expression system, Or22a neurons, which are housed in basiconic sensilla. These do not express endogenous IR8a (or tuning IRs) and, because of their larger size compared to coeloconic sensilla OSNs, are more amenable to visualisation of subcellular protein localisation. Neither EGFP:IR8a^wt^ nor EGFP:IR8a^N669Q^ localised to sensory cilia when expressed alone in Or22a neurons ([28] and data not shown), reflecting the dependence of IR8a upon a tuning IR to form transport-competent complexes. We first expressed these versions of IR8a together with IR75a. In combination with EGFP:IR8a^wt^, IR75a localised to the ciliated endings of Or22a neurons (Fig. 3a). By contrast, IR75a+EGFP:IR8a^N669Q^ was not detected beyond the inner segment (Fig. 3a). This is similar to the failure of endogenously expressed IR64a to localise with EGFP:IR8a^N669Q^ in sacculus neurons (Fig. 2b). We tested two other IR8a-depending tuning IRs, IR75c and IR84a. Both localised to sensory cilia together with EGFP:IR8a^wt^, and in contrast to IR64a or IR75a, both also localised with EGFP:IR8a^N669Q^ (Fig. 3b, c). The IR8a^N669Q^ mutant therefore reveals an unexpected heterogeneity in the cilia-targeting properties of IR complexes, with some (i.e. those containing IR64a or IR75a) critically dependent on the CREL glycosylation site, and others (i.e. those containing IR75c or IR84a) independent of this post-translational modification.

Our observation that wild-type IR8a can promote cilia transport of IR8a^N669Q^ (Fig. 2a, b) raised the question of whether a tuning IR that is targeted to cilia with IR8a^N669Q^ can facilitate the localisation of a tuning IR that cannot, if they are incorporated into a common complex. We tested this possibility in two ways: first, we examined the distribution of IR64a in its own neurons expressing EGFP:IR8a^N669Q^ (but not endogenous IR8a) together with a control receptor (IR75a, which cannot localise to cilia with IR8a^N669Q^) or test receptors (IR75c or IR84a, which can localise with IR8a^N669Q^). While co-expression of IR75c or IR84a (but not IR75a) promoted cilia targeting of EGFP:IR8a^N669Q^, in no case did this lead to localisation of IR64a to the sensory compartment (Additional file 6: Figure S6A). Rather, the levels of IR64a were substantially reduced upon co-expression of an additional tuning IR. This might be because these ectopically expressed IRs preferentially combine with EGFP:IR8a^N669Q^, thereby excluding IR64a from associating with the co-receptor resulting in its destabilisation (as observed previously [28, 32]). Second, in Or22a neurons, we misexpressed IR75a together with EGFP:IR8a^wt^ or EGFP:IR8a^N669Q^, in the absence or presence of IR75c. Unexpectedly, we observed that addition of IR75c led to lower levels and abolished cilia localisation of IR75a when co-expressed with EGFP:IR8a^wt^ (Additional file 6: Figure S6B), suggesting that IR75c outcompetes—rather than collaborates—with IR75a to form stable, transport-competent complexes. Together, these results indicate that different tuning IRs do not readily assemble into a common complex with IR8a.

We next asked what molecular features might explain why some IRs can localise with EGFP:IR8a^N669Q^. IR75c and IR84a are not distinguished from IR64a and IR75a by phylogenetic relatedness [5] or any obvious sequence motifs (data not shown). Given that it is lack of an *N*-glycosylation site on IR8a that exposes distinct properties of these tuning receptors, we hypothesised that these IRs have complementary glycosylation sites. The LBD of IR84a contains three putative *N*-glycosylation motifs (N222, N272, N289) located on the predicted external surface of the domain (based upon comparison of their location with structures of the iGluR LBD (e.g. [33])). We generated a mutant version of IR84a in which all of these sites were mutated (mCherry:IR84a^N222Q,N272Q,N289Q^) and expressed this receptor (or an mCherry:IR84a^wt^ control) together with EGFP:IR8a^wt^ or EGFP:IR8a^N669Q^ in Or22a neurons. Cilia localisation was observed in all cases (Additional file 7: Figure S7), suggesting that IR84a LBD glycosylation is not an essential compensating factor that permits localisation with IR8a^N669Q^.

### IR8a CREL and the CREL *N*-glycosylation site are important for ER export

To determine where in the endomembrane system the trafficking of IR8a^∆CREL^ and IR8a^N669Q^ is blocked, we visualised the distribution of these EGFP-tagged receptors relative to markers for different organelles: endoplasmic reticulum (ER) (labelled with tdTomato:Sec61β [34]), Golgi apparatus (labelled with γCOP:mRFP [35]) and the cilia transition zone (labelled with antibodies against B9d1 [36]). We used genetically encoded markers for the ER and Golgi in order to express them only in the OSNs of interest, thereby avoiding confounding signal from the organelle-rich epidermal cells in the antenna [37].

**Fig. 4:**
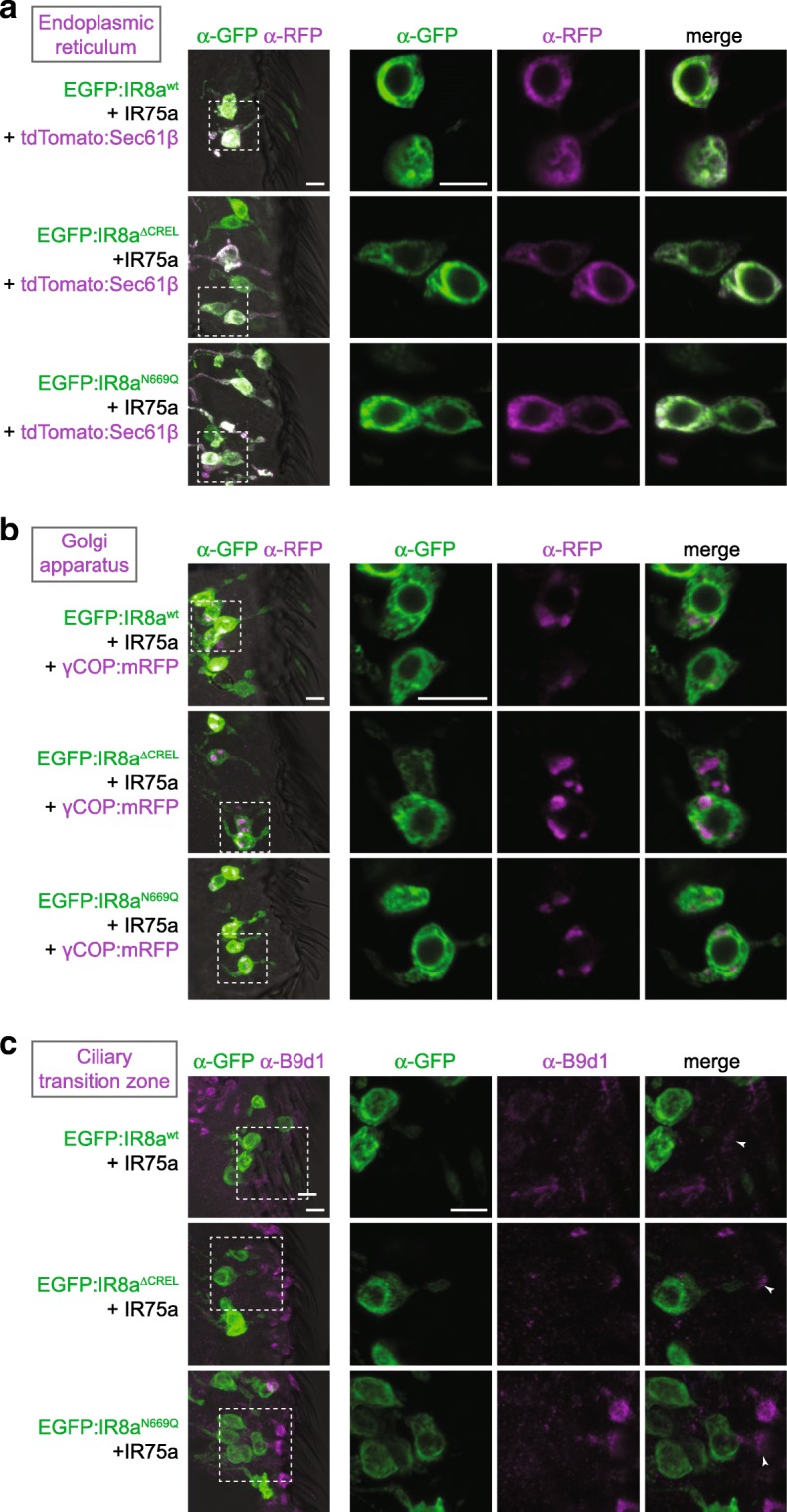
The IR8a CREL and CREL *N*-glycosylation site are important for ER export. **a** Immunofluorescence with antibodies against GFP (green) and RFP/Tomato (magenta) on antennal sections of animals expressing the indicated transgenes in Or22a neurons. The images on the right are high-magnification, single optical slices taken within the region shown in the lower-magnification view on the left in this and subsequent panels. Genotypes are of the form *UAS-EGFP:Ir8a*^*x*^*/UAS-Ir75a;Or22a-Gal4/UAS-tdTomato:Sec61β*. Scale bars: 5 μm. For each genotype, the phenotype was assessed in multiple sections of antennae from at least 20 animals from two independent genetic crosses, in this and the following panels. **b** Immunofluorescence with antibodies against GFP (green) and RFP (magenta) on antennal sections of animals expressing the indicated transgenes in Or22a neurons. Genotypes are of the form *UAS-EGFP:Ir8a*^*x*^*/UAS-Ir75a;Or22a-Gal4/UAS-γCOP:mRFP*. Scale bars: 5 μm. **c** Immunofluorescence with antibodies against GFP (green) and B9d1 (magenta) on antennal sections of animals expressing the indicated transgenes in Or22a neurons. Genotypes are of the form *UAS-EGFP:Ir8a*^*x*^*/UAS-Ir75a;Or22a-Gal4/+*. Scale bars: 5 μm.

We first analysed the distribution of EGFP:IR8a variants—co-expressed with IR75a—in Or22a neurons (Fig. 4). In these cells, the ER marker displayed a prominent perinuclear signal, but also extended up to the base of the sensillar hair (Fig. 4a), suggesting that this organelle is broadly distributed in OSNs. EGFP:IR8a^wt^ had a similar, though not identical, distribution in the soma and inner dendrite, in addition to its terminal localisation in the sensory cilia (where intracellular organelles are not observed). Both EGFP:IR8a^∆CREL^ and EGFP:IR8a^N669Q^, though absent from the cilia, displayed a similar overlap with the ER (Fig. 4a). The Golgi marker was found in a few large puncta present primarily in the OSN soma (Fig. 4b). These puncta were almost entirely devoid of EGFP:IR8a^wt^ (Fig. 4b), suggesting that IRs transit rapidly through this organelle. Alternatively, given the broader distribution of the ER (Fig. 4a), the receptors might follow a Golgi-independent route from the ER to the sensory compartment, as described for other cilia membrane proteins [38]. Regardless, neither EGFP:IR8a^∆CREL^ nor EGFP:IR8a^N669Q^ displayed Golgi localisation, indicating that their inability to localise to sensory cilia is not due to transport arrest in the Golgi. Similarly, we did not detect overlap of any EGFP:IR8a variant and the transition zone marker (Fig. 4c), indicating that the wild-type protein passages quickly from the inner to outer dendrite and that the mutant proteins do not become blocked at this stage in their transport to cilia. We repeated these analyses in IR64a-expressing sacculus neurons and made very similar observations (Additional file 8: Figure S8): EGFP:IR8a^∆CREL^ and EGFP:IR8a^N669Q^overlapped with the ER marker, but not Golgi or transition zone markers. Together, these observations are consistent with a model in which the IR8a^∆CREL^ and IR8a^N669Q^ fail to localise to sensory cilia because they are trapped in the ER, rather than in later compartments of the transport pathway.

### IR8a CREL *N*-glycosylation is dispensable for the function of IR complexes

**Fig. 5:**
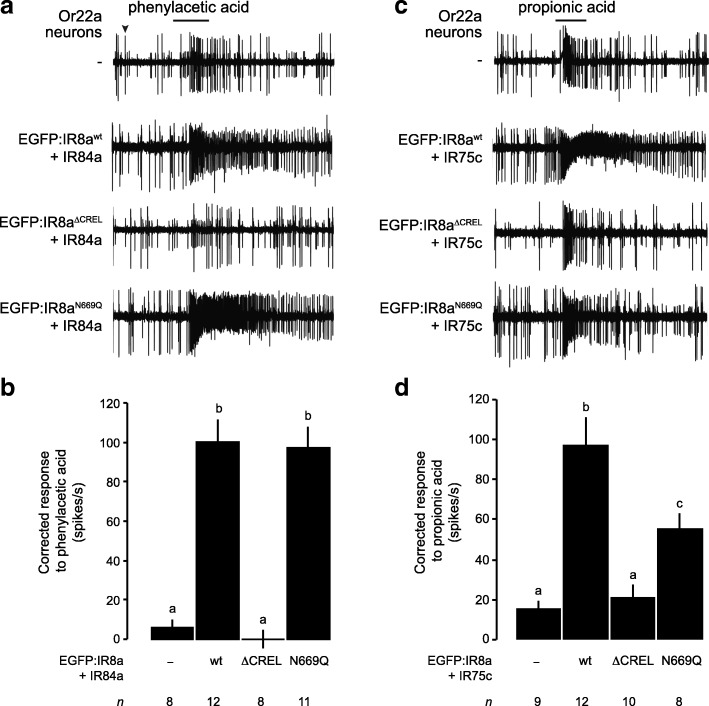
The IR8a CREL *N*-glycosylation site is not essential for odour-evoked IR signalling. **a** Representative traces of the responses of Or22a neurons—those exhibiting the larger of the two spike amplitudes within this sensillum (black arrowhead)—expressing the indicated combinations of IRs, exposed to a 1-s pulse (black bar) of phenylacetic acid (1% *v*/*v*). Genotypes are of the form *UAS-EGFP:Ir8a*^*x*^/*UAS-Ir84a;Or22a-Gal4/+*, except for the control (*Or22a-Gal4/+*). **b** Quantification of the odour-evoked responses of the genotypes shown in **a**. Mean solvent corrected responses ±SEM are shown (*n* (number of sensilla) are indicated beneath each bar; mixed genders). Bars labelled with different letters are statistically different from each other (*p* < 0.05; Student’s *t* test with Benjamini and Hochberg correction for multiple comparisons). **c** Representative traces of the responses of Or22a neurons expressing the indicated combinations of IRs, exposed to a 1-s pulse (black bar) of propionic acid (1% *v*/*v*). Genotypes are of the form *UAS-EGFP:Ir8a*^*x*^/+;*Or22a-Gal4/UAS-Ir75c*, except for the control (*Or22a-Gal4/+*). **d** Quantification of the odour-evoked responses of the genotypes shown in **c**, presented as in **b**

The localisation of a subset of IRs to sensory cilia with IR8a^N669Q^ allowed us to ask whether this *N*-glycosylation site is important for odour-evoked signalling. Using single sensilla electrophysiological recordings, we measured the olfactory responses of Or22a neurons co-expressing the EGFP-tagged IR8a variants with IR84a upon stimulation with phenylacetic acid, the best-known agonist of this tuning receptor [39]. IR84a+EGFP:IR8a^wt^ conferred robust responses to this odour compared to control neurons that do not express these receptors (Fig. 5a, b). Consistent with the lack of cilia localisation, IR84a+EGFP:IR8a^∆CREL^ did not increase responses above background levels (Fig. 5a, b). By contrast, IR84a+EGFP:IR8a^N669Q^-expressing neurons exhibited phenylacetic acid sensitivity indistinguishable from that conferred by IR84a+EGFP:IR8a^wt^ (Fig. 5a, b).

We extended this analysis with a second tuning receptor, IR75c, which detects propionic acid [11]. Although Or22a neurons displayed weak endogenous responses to this odour, these are much lower than those exhibited upon expression of IR75c+EGFP:IR8a^wt^ (Fig. 5c, d). As expected, IR75c+EGFP:IR8a^∆CREL^-expressing neurons had similar propionic acid sensitivity to those lacking IRs (Fig. 5c, d). In contrast to the observations with IR84a, IR75c+EGFP:IR8a^N669Q^ co-expression yielded responses that are decreased compared to IR75c+EGFP:IR8a^wt^ (Fig. 5c, d), which might reflect defects in function or diminished levels of cilia localisation not discernable with available reagents. Together, however, these observations indicate that the CREL glycosylation is not essential for IR signalling.

### The IR8a CREL is likely to be exposed on an external face of an IR heterotetrameric complex

**Fig. 6:**
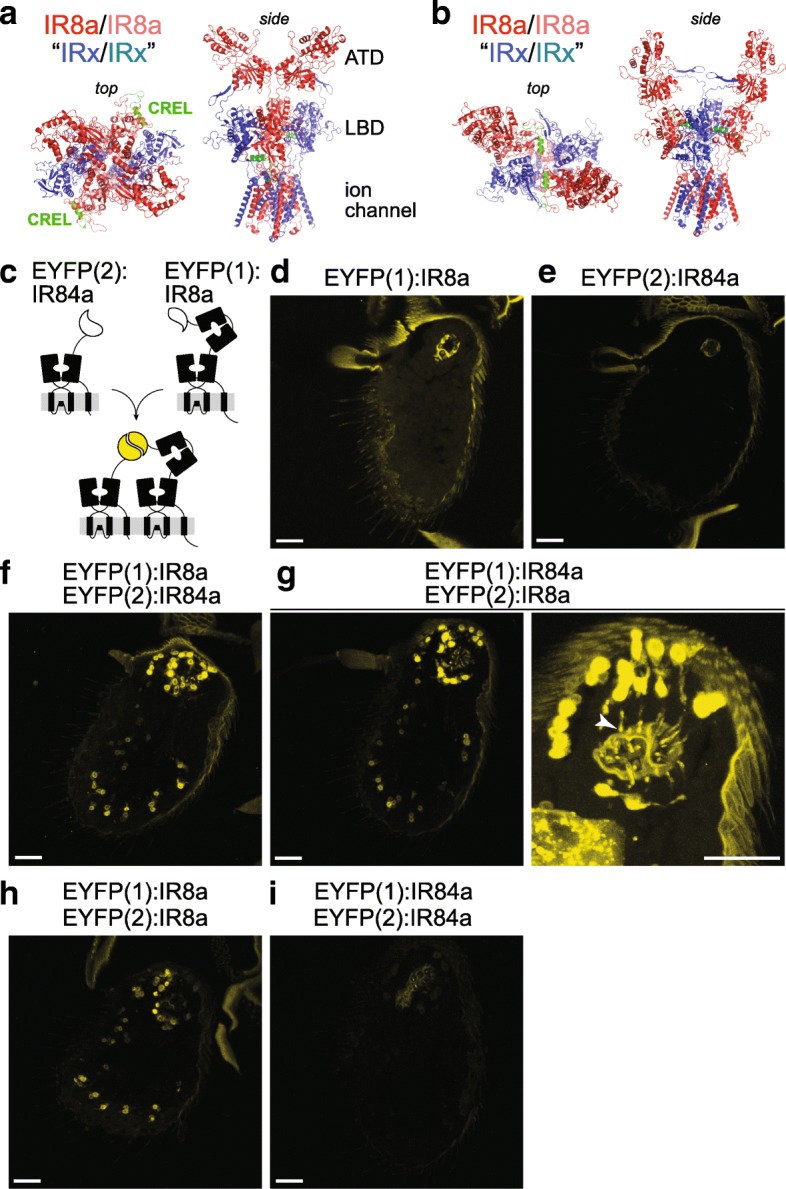
Location of the CREL in a heterotetrameric IR complex model. **a**, **b** Two hypothetical configurations of a heterotetramer of two IR8a subunits (dark/pale red) and two tuning IR subunits (dark/pale blue), in which the IR8a ATDs have **a** proximal (contacting) or **b** distal (non-contacting) positions. Top and side views are shown in slightly different orientations to facilitate visualisation of the IR8a CREL (green). The structure was built through coarse-grained homology modelling of *D. melanogaster* IR8a on the homotetrameric GluA2 structure [33]; the IR tuning subunits are represented simply by the same IR8a model from which the ATD is deleted. **c** Schematic of the principle of EYFP reconstitution through complex formation and/or close proximity of EYFP fragment:IR fusion proteins. **d–i** Endogenous EYFP fluorescence in antennal sections of animals expressing the indicated combinations of EYFP fragment fusions in Ir8a neurons. The higher magnification sacculus image in **g** (right) reveals the cilia localisation of fluorescent signals (arrowhead); here, the gain setting during imaging was increased, resulting in higher cuticular autofluorescence. Genotypes: **d** *UAS-EYFP(1):Ir8a/+;Ir8a-Gal4/+*, **e** *UAS-EYFP(2):Ir84a/+;Ir8a-Gal4/+*, **f** *UAS-EYFP(1):Ir8a/UAS-EYFP(2):Ir84a;Ir8a-Gal4/+*, **g** *UAS-EYFP(1):Ir84a/UAS-EYFP(2):Ir8a;Ir8a-Gal4/+*, **h** *UAS-EYFP(1):Ir8a/UAS-EYFP(2):Ir8a;Ir8a-Gal4/+* and **i** *UAS-EYFP(1):Ir84a/UAS-EYFP(2):Ir84a;Ir8a-Gal4/+*. All scale bars: 20 μm. For each genotype, the phenotype was assessed in multiple sections of antennae from at least 20 animals from two independent genetic crosses

Finally, we explored where the CREL is likely to be located within an IR complex. Based upon our in vivo evidence (Fig. 2), subunit counting analysis in vitro [28], and by analogy with the (hetero)tetrameric stoichiometry of iGluRs [2, 3, 30], we reasoned that IR complexes are composed of two IR8a subunits and two tuning subunits. In iGluRs, the extracellular domains of the four subunits form a twofold axis of symmetry, comprising two with ‘proximal’ ATDs, which contact each other across the axis of symmetry, and two with ‘distal’ ATDs, which do not [40]. The presence of an ATD in IR8a (and IR25a) but not in tuning IRs led us to hypothesise that the IR co-receptor subunits correspond to the iGluR subunits whose ATDs interact. To examine the approximate location of the CREL in such a subunit arrangement, we generated a homology model of a putative IR complex, based upon a structure of the mammalian AMPA receptor GluA2 [33]. In the subunit configuration where IR8a ATDs interact, the CREL is exposed on the external face of the assembled LBDs (Fig. 6a). In an alternative model, where IR8a corresponds to iGluR subunits whose ATDs do not contact each other, the CREL is predicted to be buried within the interface between co-receptor and tuning IR subunits (Fig. 6b). This latter configuration seems unlikely for two reasons: first, the relatively short (and highly divergent) *N*-termini of tuning IRs may provide little or no opportunity for specific intersubunit interactions to occur within the upper layer of the complex, a region that is key for selective assembly of iGluRs [30, 41]. Second, the externally exposed IR8a CREL in the former configuration (Fig. 6a) would permit greater access to both the *N*-glycosylation machinery and to (unknown) ER export quality control sensors.

To obtain experimental evidence for the configuration of IRs in vivo, we used a protein fragment complementation assay with an enhanced yellow fluorescent protein (EYFP) reporter [42, 43]. Complementary and non-associating subfragments of EYFP (EYFP(1) and EYFP(2)) were fused to the *N*-termini of IR8a and IR84a, respectively, separated by short flexible linkers (Fig. 6c), and these proteins were expressed in Ir8a neurons singly or together. Neither fusion protein alone was fluorescent (Fig. 6d, e), but upon co-expression, we detected a robust EYFP signal in all Ir8a neurons (Fig. 6f), indicating direct association or close apposition of IR84a and IR8a. Similar results were obtained when EYFP fragment tags were exchanged on these receptors (Fig. 6g). EYFP fluorescence was detected both around the nucleus in the soma and in the ciliated dendritic endings, but not axons, indicating that the complex is likely to form in the endoplasmic reticulum (Fig. 6g, right).

In addition to analysis of these heteromeric interactions, we expressed together EYFP(1):IR8a and EYFP(2):IR8a. These fusion proteins also reconstituted EYFP fluorescence (Fig. 6h), suggesting the existence of homomeric interactions between two IR8a subunits. We also co-expressed EYFP(1):IR84a and EYFP(2):IR84a, but observed that these did not reconstitute a fluorescence signal (Fig. 6i). The simplest explanation for this result is that the EYFP fragments on IR84a subunits are not sufficiently close to each other in a tetrameric complex and/or are sterically inhibited from associating due to a ‘barrier’ of the interacting IR8a ATDs. Importantly, the result also provides a negative control that indicates that the EYFP reconstitution observed in the tagged IR84a+IR8a and IR8a+IR8a pairs is likely due to the formation of specific protein complexes, rather than simply their coexistence in the same neuronal membranes. Together, these observations are consistent with the model of an IR heterotetramer in which the ATDs of two IR8a subunits are directly apposed (Fig. 6a).

## Discussion

The characterisation of the IR8a CREL has revealed a critical role of an *N*-glycosylation site in the IR co-receptor LBD in regulating receptor transport from the ER to sensory cilia. This property is likely to be relevant for the functionally diverse IR8a- and IR25a-containing complexes in different sensory systems. Our data also provide insights into the stoichiometry and assembly of IR complexes in vivo, supporting a model in which two co-receptor subunits form a ‘core’—possibly interacting via their ATDs [28]—with which two tuning IR subunits associate in the ER. Future structural analysis of IR8a (and other IRs) will be necessary to uncover the precise conformation of the CREL within IR complexes. Outside of this sequence, the IR8a LBD contains several additional predicted *N*-glycosylation sites; while their role (if any) is unknown, our data indicate that *N*-glycosylation of the CREL has a unique contribution to the regulation of IR trafficking.

The unexpected heterogeneity in the localisation properties of different IR complexes in which *N*-glycosylation of the CREL is prevented suggests that each tuning/co-receptor complex has a unique conformation that is assessed at a trafficking checkpoint in the ER: in some cases, the CREL *N*-glycosylation site is a key part of the signal permitting exit from this organelle, while in others it is dispensable. There are no obvious sequence motifs that can account for the distinction of these different types of IRs. We speculate that the observed heterogeneity is related to the conformational flexibility of the LBDs within an IR complex, as this is a property of iGluR LBDs that influences ER export [44], and tuning IR LBDs are highly diverse in sequence. These context-dependent trafficking properties in the IR family are reminiscent of the variable dependence of different mammalian odorant receptors on specific accessory proteins for ER exit [45]. Such heterogeneity may reflect the ‘conflict’ that exists during the diversification of chemosensory receptor families, as individual members are under selective pressure both to maintain conserved cellular properties (i.e. trafficking to sensory cilia) and to evolve novel sensory-detection capacities.

## Conclusions

Our data reveal an important role for the IR co-receptor LBD in control of intracellular transport, provide novel insights into the stoichiometry and assembly of IR complexes and uncover an unexpected heterogeneity in the trafficking regulation of this sensory receptor family.

## Methods

### Bioinformatics

Alignments were made with MUSCLE [46] and visualised in Jalview 2.9.0b2 [47]. Secondary structure predictions were made using Quick2D [48]. The IR8a homology model was built using SWISS-MODEL [49], with the *R. norvegicus* GluA2 structure (PDB: 6DLZ [33]) as template.

### Molecular biology

Deletions and point mutations in *Ir8a* and *Ir84a* coding sequences (lacking the region encoding the endogenous signal sequence, as described previously [28]) were introduced by standard PCR-based mutagenesis methods. Wild-type and mutant *Ir8a* sequences were subcloned into *pUAST-EGFP* *attB*, which encodes the calreticulin signal sequence fused to EGFP [28]. Wild-type and mutant *Ir84a* sequences were subcloned into an equivalent *pUAST-mCherry* *attB* [28]. Similarly, transgenes for EYFP protein fragment complementation were generated by joining sequences encoding EYFP(1) or EYFP(2) (with the calreticulin signal sequence) [43] to *Ir8a* or *Ir84a* sequences with an intervening short linker (encoding [GGGGS]_2_) in *pUAST attB* [50].

### Biochemistry

#### Protein purification

The sequence of *ZnevIr8a*^*S1S2*^ was synthesised by GENEWIZ and encodes the S1 domain (residues A366-P490) and the S2 domain (residues P608-N781) connected by a GTGT peptide. This sequence was cloned into a pcDNA vector for mammalian cell expression with a human IL2 signal sequence (MYRMQLLSCIALSLALVTNS), a 9xHis-tag and a Factor Xa cleavage site added to its *N*-terminus. The protein was expressed and secreted from FreeStyle HEK 293-F cells (Thermo Fisher) and purified directly from the cell culture medium using Ni^2+^-NTA resins. The protein was eluted from the resins with a high concentration of imidazole and further purified by a Superdex-200 size-exclusion column in a buffer containing 50 mM Tris, pH 8.0 and 400 mM NaCl.

#### Peptide sequencing by LC-MS/MS

Tryptic peptides were analysed by LC-MS/MS using an LTQ-Orbitrap XL MS (Thermo Fisher) coupled on-line with an Easy-nLC 1000 (Thermo Fisher) [51]. Each MS/MS experiment consisted of one MS scan in FT mode (350–1800 m/z, resolution of 60,000 at m/z 400) followed by ten data-dependent MS/MS scans in IT mode with normalised collision energy at 29%. Protein identification and characterisation were performed by database searching using the Batch-Tag within the developmental version of Protein Prospector (v5.17.0) [51] against a targeted database consisting of *Znev*IR8a sequences. The mass accuracies for parent ions and fragment ions were set as ± 20 ppm and 0.6 Da, respectively. Trypsin was set as the enzyme, and a maximum of two missed cleavages were allowed. Protein *N*-terminal acetylation, methionine oxidation, *N*-terminal conversion of glutamine to pyroglutamic acid and asparagine deamidation were set as variable modifications. Peptide relative abundances were evaluated based on extracted chromatograms of the selected ions during MS scans.

### *Drosophila* strains

Flies were maintained on a standard corn flour, yeast and agar medium at 25 °C in 12-h light:12-h dark conditions. The wild-type strain was *w*^*1118*^. We used the following published *D. melanogaster* strains: *Ir8a*^*1*^ (RRID:BDSC_41744) [28], *Ir8a-Gal4* (RRID:BDSC_41731) [28], *Or22a-Gal4* (RRID:BDSC_9952) [52], *UAS-Ir75a* [10], *UAS-Ir75c* [11], *UAS-Ir84a* (RRID:BDSC_41740) [1], *UAS-tdTomato:Sec61β* (BSDC_64747) [34] and *UAS-γCOP:mRFP* (BDSC_29714) [35]. New transgenic flies were generated by Genetic Services Inc. or BestGene Inc., via the phiC31 site-specific integration system, using the *attP40* and *attP2* landing site strains [53] for insertions on chromosomes 2 and 3, respectively. Genotypes are provided in the figure legends. Both sexes were used in most experiments except for some genotypes containing the *Ir8a*^*1*^ mutant allele (on the X chromosome), when only hemizygous males (*Ir8a*^*1*^*/Y*) were used; there is no known sexually dimorphic expression or function of IR8a.

### Immunohistochemistry and imaging

The following primary antibodies were used: guinea pig anti-IR8a (1:1000) (RRID:AB_2566833) [28], rabbit anti-IR64a (1:1000) (RRID:AB_2566854) [12], rabbit anti-IR75a (1:1000) (RRID: AB_2631091) [10], rabbit anti-IR75c (1:200) (RRID: AB_2631094) [11], guinea pig anti-B9d1 (1:2500) [36], mouse monoclonal 21A6 (1:200) (RRID:AB_528449) (Developmental Studies Hybridoma Bank), mouse anti-GFP (1:1000) (Invitrogen A11120), anti-RFP (1:1000) (Abcam ab62341). Secondary antibodies: Alexa488 anti-mouse (1:1000) (Invitrogen A11029), Cy3 anti-guinea pig (1:1000) (Jackson Immunoresearch 106–166-003), Cy3 anti-rabbit (1:1000) (MILAN Analytica AG 111–165-144 0), Cy5 anti-guinea pig (1:1000) (Abcam ab102372).

Microscopy was performed using a Zeiss LSM 710 Inverted Laser Scanning Confocal Microscope. Confocal images were processed with Fiji [54].

For all experiments presented, images of antennal sections are representative of analysis of a minimum of 20 flies from at least two independent genetic crosses (samples sizes and number of replicates are provided in the corresponding figure legends). The phenotypes described are generally qualitative in nature (e.g. localisation or no localisation to cilia). It is very difficult to accurately quantify protein levels in cilia because the signals are inherently variable within a sample due, for example, to heterogeneous driver strength and the precise section cut, which can influence antibody permeation of the sensillar hair. Moreover, the cilia signals cannot be confidently normalised to the expression level in the corresponding soma, as these two parts of the neuron may not necessarily be in the same tissue section, and the dendritic region typically has a very weak signal, which leads to ambiguity in determining the soma corresponding to a given cilium.

### Electrophysiology

Single sensillum extracellular recordings were performed and analysed essentially as described [55], using odour cartridges assembled as detailed in [56]. Phenylacetic acid (CAS #103-82-2) and propionic acid (CAS #79-09-4) were from Sigma-Aldrich and were of the highest purity available. Odorants were used at 1% (*v*/*v*) in double-distilled H_2_O.

## Additional files

Additional file 1: **Figure S1.** Alignment of IR and iGluR LBDs. Multiple sequence alignment of the predicted LBD sequence from the indicated *Rattus norvegicus* iGluRs and *Drosophila melanogaster* IRs. The approximate position of the CREL is indicated, and the conserved *N*-glycosylation site within this sequence is highlighted with a red box. (PDF 3827 kb)

Additional file 2: **Figure S2.** IR25a CREL alignment. Alignment of the protein sequence spanning the CREL in IR25a orthologues from the indicated species. Predicted *N*-glycosylation sites are highlighted with red boxes and predicted secondary structure is shown below the alignment. Species (top-to-bottom): *Drosophila melanogaster*, *Drosophila simulans*, *Drosophila ananassae*, *Drosophila willistoni*, *Drosophila grimshawi*,* Anopheles gambiae*, *Aedes aegypti, Culex quinquefasciatus, Bombyx mori*, *Camponotus floridanus*, *Apis mellifera*, *Nasonia vitripennis* (two orthologues), S*olenopsis invicta*, *Tribolium castaneum, Acyrthosiphon pisum*, *Pediculus humanus*, *Zootermopsis nevadensis, Schistocerca gregaria*, *Phyllium siccifolium*, *Thermobia domestica*, *Lepismachilis y-signata*, *Daphnia pulex*, *Panulirus argus*, *Limulus polyphemus* (two orthologues), *Metaseiulus occidentalis*, *Caenorhabditis elegans*, *Capitella capitata*, *Aplysia californica*, *Crassostrea gigas*, *Lottia gigantea* (two orthologues). (PDF 2716 kb)

Additional file 3: **Figure S3.** The IR8a CREL contains a single *N*-linked glycosylation site. (A) Top: extracted ion chromatograms of a *Zootermopsis nevadensis* (*Znev*) IR8a tryptic peptide containing a deamidated asparagine (N*) (m/z 648.2984^2+^) before and after PNGase F treatment; the abundance of this peptide increases 1000-fold after treatment. Bottom: MS/MS spectrum identifying the corresponding peptide (DITLN*SSSDQSK, which is located within the CREL (Fig. 1b)). (B) Top: extracted ion chromatograms of a *Znev*IR8a tryptic peptide containing a deamidated asparagine (m/z 676.3276^2+^) before and after PNGase F treatment Bottom: MS/MS spectrum identifying the corresponding peptide sequence (N*AEDVLYNVWK), which lies at the beginning of the CREL sequence (Fig. 1b). In this peptide, the deamidated terminal asparagine is not indicative of an *N*-glycosylated residue, because peptide abundance is similar with and without PNGase F treatment, and most likely reflects an artefact of MS sample preparation. (PDF 264 kb)

Additional file 4: **Figure S4.** IR8a^∆CREL^ and IR8a^N669Q^ are not destabilised in young animals. Immunofluorescence with antibodies against GFP (green), IR8a (blue) and IR64a (red) on antennal sections of animals (< 1 day old) expressing the indicated transgenes in Ir8a neurons in an *Ir8a* mutant background. Scale bar: 10 μm. Genotypes are of the form: *Ir8a*^*1*^*/Y;Ir8a-Gal4/UAS-EGFP:Ir8a*^*x*^. (PDF 6725 kb)

Additional file 5: **Figure S5.** Heterogeneous localisation properties of IR8a^N669Q^ in coeloconic sensilla. (A) Immunofluorescence with antibodies against GFP (green) and IR8a (magenta) on antennal sections of animals expressing the indicated transgenes in Ir8a neurons. Genotypes are of the form: *Ir8a-Gal4/UAS-EGFP:Ir8a*^*x*^. Arrowheads mark examples of sensilla in which receptors are detected in the OSN cilia; this was determined by overlaying the fluorescence signal onto a bright-field channel, as shown in the merged images. EGFP:IR8a^∆CREL^ does not traffic beyond the inner segment. Scale bar (for all panels in this figure): 10 μm. For each genotype, the phenotype was assessed in multiple sections of antennae from at least 20 animals from two independent genetic crosses. (B) Immunofluorescence with antibodies against GFP (green) and IR8a (magenta) on antennal sections of animals expressing the indicated transgenes in Ir8a neurons in an *Ir8a* mutant background. Genotypes are of the form: *Ir8a*^*1*^*/Y;Ir8a-Gal4/UAS-EGFP:Ir8a*^*x*^. Arrowheads mark examples of sensilla in which receptors are detected in the OSN cilia. For each genotype, the phenotype was assessed in multiple sections of antennae from at least 20 animals from two independent genetic crosses. (PDF 8558 kb)

Additional file 6: **Figure S6.** Tuning IRs compete for, rather than assemble together with, IR8a^N669Q^. (A) Immunofluorescence with antibodies against GFP (green) and IR64a (magenta) on antennal sections of animals expressing the indicated transgenes in Ir8a neurons in an *Ir8a* mutant background. Genotypes are of the form: *Ir8a*^*1*^*/Y;UAS-EGFP:Ir8a*^*N669Q*^*/UAS-IrXX;Ir8a-Gal4/+*. The white asterisks in the right-hand panels indicate the central cavity of sacculus chamber 3 into which the OSN ciliated dendrites project. Due to the weak expression of IR64a in these tissues (compared to, for example, Fig. 2a), the gain setting during imaging was increased, resulting in high cuticular autofluorescence in the magenta channel, which reveals both the antennal surface and the lining of the sacculus. The arrowheads in the left-hand panels mark the ciliated endings of neurons containing EGFP:IR8a^N669Q^ (but not IR64a). Scale bar (for all panels in this figure): 10 μm. For each genotype, the phenotype was assessed in multiple sections of antennae from at least 20 animals from two independent genetic crosses. (B) Immunofluorescence with antibodies against GFP (green) and IR75a (magenta) on antennal sections of animals expressing the indicated transgenes in Or22a neurons. Genotypes are of the form: *UAS-EGFP:Ir8a*^*x*^*/+;Or22a-Gal4/UAS-Ir75a* (top two rows) and *UAS-EGFP:Ir8a*^*x*^*/UAS-Ir75c;Or22a-Gal4/UAS-Ir75a* (bottom two rows). For each genotype, the phenotype was assessed in multiple sections of antennae from at least 20 animals from two independent genetic crosses. (PDF 12021 kb)

Additional file 7: **Figure S7.** Predicted IR84a LBD *N*-glycosylation sites are not essential for cilia localisation of IR complexes in the presence or absence of IR8a CREL *N*-glycosylation. Immunofluorescence with antibodies against GFP (green) and RFP (magenta) on antennal sections of animals expressing the indicated transgenes in Or22a neurons. Genotypes are of the form: *UAS-EGFP:Ir8a*^*x*^*/+;Or22a-Gal4/UAS-mCherry:Ir84a*^*x*^. Scale bar (for all panels in this figure): 10 μm. For each genotype, the phenotype was assessed in multiple sections of antennae from at least 30 animals from three independent genetic crosses. (PDF 4768 kb)

Additional file 8: **Figure S8.** The IR8a CREL and the CREL *N*-glycosylation site are important for ER export. (A) Immunofluorescence with antibodies against GFP (green) and RFP/Tomato (magenta) on antennal sections of animals expressing the indicated transgenes in Ir8a sacculus neurons. The images on the right are high-magnification, single optical slices taken within the region shown in the lower-magnification view on the left, in this and the following panels. Genotypes are of the form: *Ir8a*^*1*^*/Y;Ir8a-Gal4/UAS-EGFP:Ir8a*^*x*^*;UAS-tdTomato:Sec61β/+*. Scale bars: 5 μm. For each genotype, in this and the following panels, the phenotype was assessed in multiple sections of antennae from at least 20 animals from two independent genetic crosses. (B) Immunofluorescence with antibodies against GFP (green) and RFP (magenta) on antennal sections of animals expressing the indicated transgenes in Ir8a neurons. Genotypes are of the form: *Ir8a*^*1*^*/Y;Ir8a-Gal4/UAS-EGFP:Ir8a*^*x*^*;UAS-γCOP:mRFP/+*. Scale bars: 5 μm. (C) Immunofluorescence with antibodies against GFP (green) and B9d1 (magenta) on antennal sections of animals expressing the indicated transgenes in Ir8a neurons. Genotypes are of the form: *Ir8a*^*1*^*/Y;Ir8a-Gal4/UAS-EGFP:Ir8a*^*x*^. Scale bars: 5 μm. (PDF 9867 kb)
