# Supplementary figures and images for: In vivo assembly and trafficking of olfactory Ionotropic Receptors
Source: BMC Biol. 2019 Apr 17;17:34. doi: 10.1186/s12915-019-0651-7 (PMC6472016; doi:10.1186/s12915-019-0651-7)

Figure S1

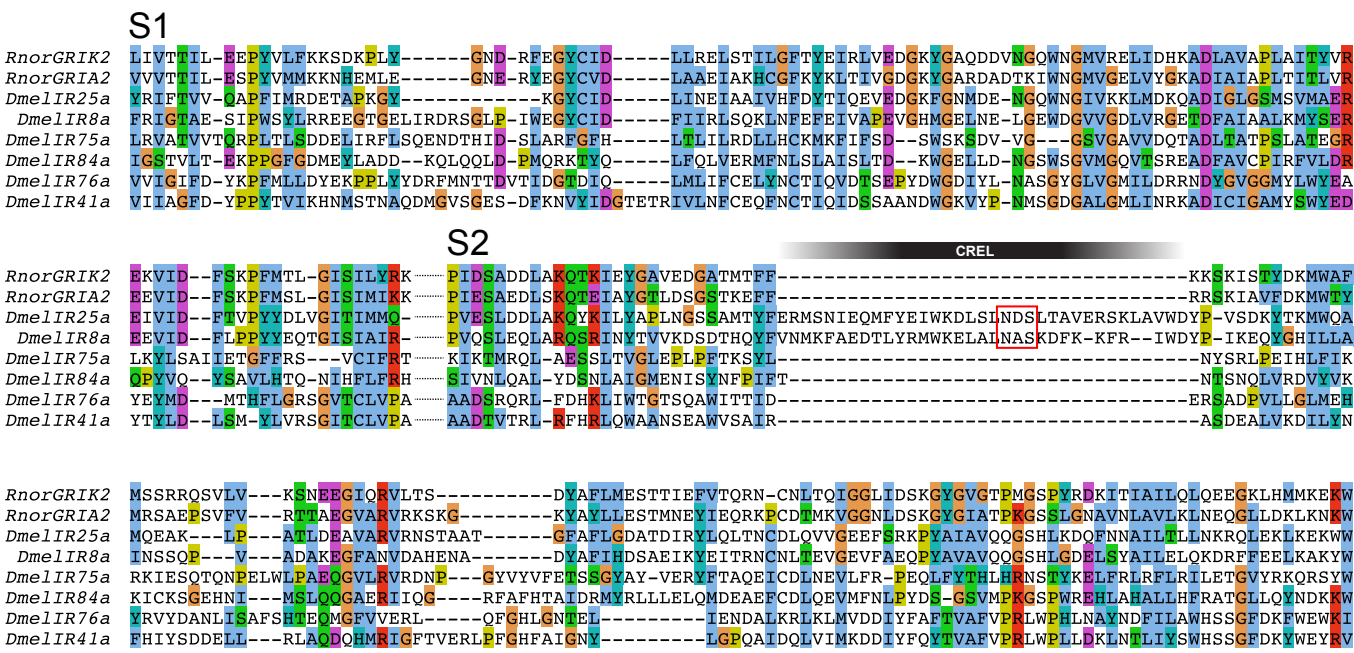

Supplement: Supplementary file 1 — Figure S1. Alignment of IR and iGluR LBDs. Multiple sequence alignment of the predicted LBD sequence from the indicated Rattus norvegicus iGluRs and Drosophila melanogaster IRs. The approximate position of the CREL is indicated, and the conserved N-glycosylation site within this sequence is highlighted with a red box. (PDF 3827 kb) [file 12915_2019_651_MOESM1_ESM.pdf]

Figure S2

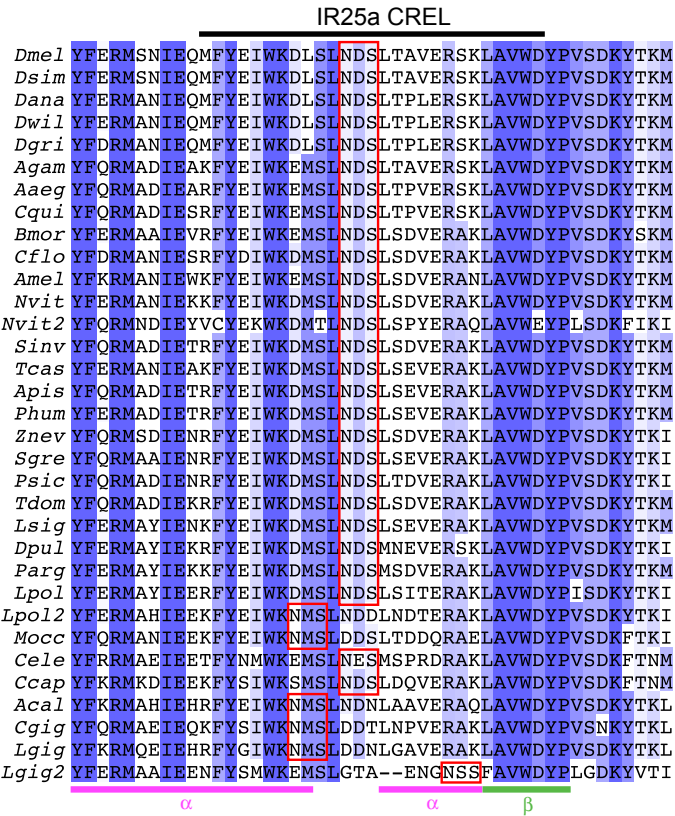

Supplement: Supplementary file 2 — Figure S2. IR25a CREL alignment. Alignment of the protein sequence spanning the CREL in IR25a orthologues from the indicated species. Predicted N-glycosylation sites are highlighted with red boxes and predicted secondary structure is shown below the alignment. Species (top-to-bottom): Drosophila melanogaster, Drosophila simulans, Drosophila ananassae, Drosophila willistoni, Drosophila grimshawi, Anopheles gambiae, Aedes aegypti, Culex quinquefasciatus, Bombyx mori, Camponotus floridanus, Apis mellifera, Nasonia vitripennis (two orthologues), Solenopsis invicta, Tribolium castaneum, Acyrthosiphon pisum, Pediculus humanus, Zootermopsis nevadensis, Schistocerca gregaria, Phyllium siccifolium, Thermobia domestica, Lepismachilis y-signata, Daphnia pulex, Panulirus argus, Limulus polyphemus (two orthologues), Metaseiulus occidentalis, Caenorhabditis elegans, Capitella capitata, Aplysia californica, Crassostrea gigas, Lottia gigantea (two orthologues). (PDF 2716 kb) [file 12915_2019_651_MOESM2_ESM.pdf]

Figure S3

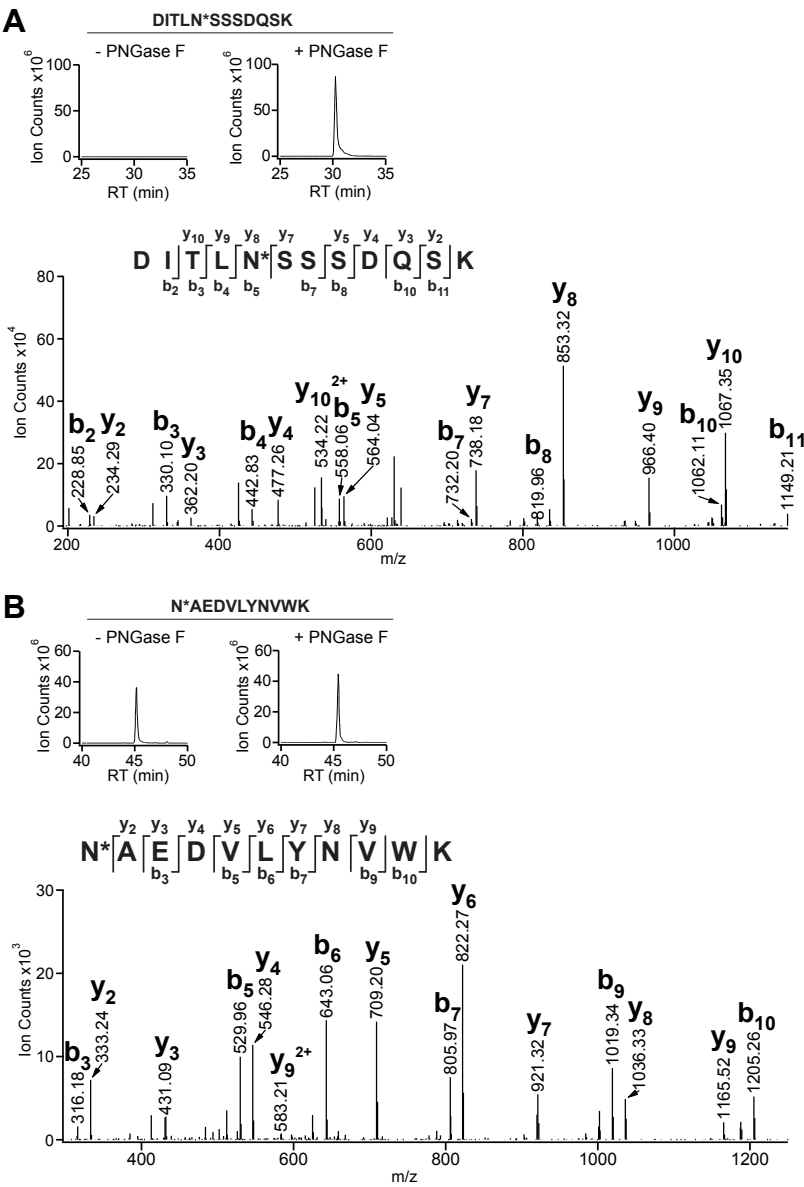

Supplement: Supplementary file 3 — Figure S3. The IR8a CREL contains a single N-linked glycosylation site. (A) Top: extracted ion chromatograms of a Zootermopsis nevadensis (Znev) IR8a tryptic peptide containing a deamidated asparagine (N*) (m/z 648.29842+) before and after PNGase F treatment; the abundance of this peptide increases 1000-fold after treatment. Bottom: MS/MS spectrum identifying the corresponding peptide (DITLN*SSSDQSK, which is located within the CREL (Fig. 1b)). (B) Top: extracted ion chromatograms of a ZnevIR8a tryptic peptide containing a deamidated asparagine (m/z 676.32762+) before and after PNGase F treatment Bottom: MS/MS spectrum identifying the corresponding peptide sequence (N*AEDVLYNVWK), which lies at the beginning of the CREL sequence (Fig. 1b). In this peptide, the deamidated terminal asparagine is not indicative of an N-glycosylated residue, because peptide abundance is similar with and without PNGase F treatment, and most likely reflects an artefact of MS sample preparation. (PDF 264 kb) [file 12915_2019_651_MOESM3_ESM.pdf]

Figure S4

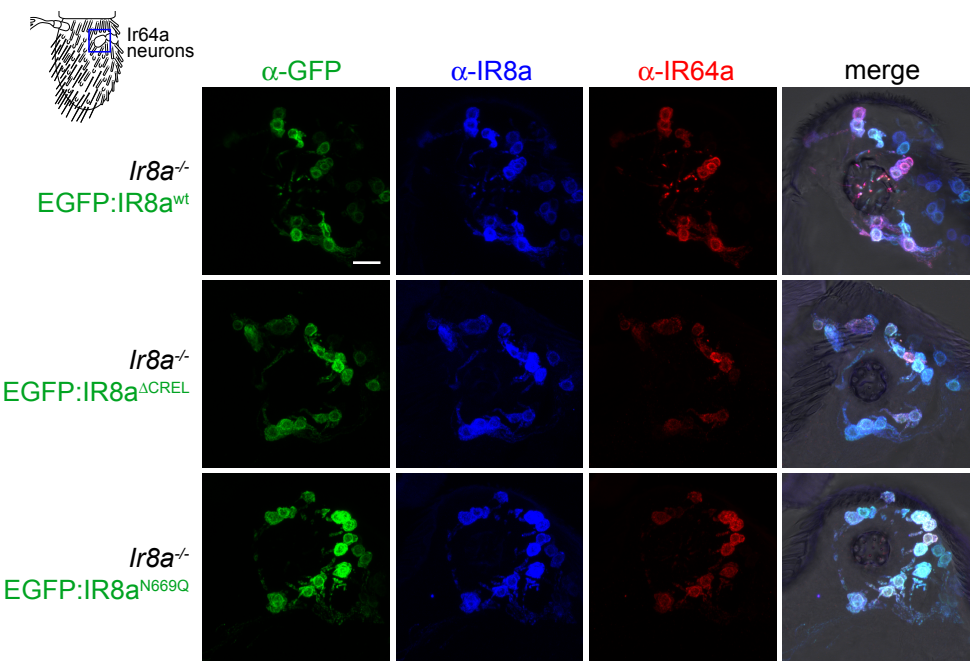

Supplement: Supplementary file 4 — Figure S4. IR8a∆CREL and IR8aN669Q are not destabilised in young animals. Immunofluorescence with antibodies against GFP (green), IR8a (blue) and IR64a (red) on antennal sections of animals (< 1 day old) expressing the indicated transgenes in Ir8a neurons in an Ir8a mutant background. Scale bar: 10 μm. Genotypes are of the form: Ir8a1/Y;Ir8a-Gal4/UAS-EGFP:Ir8ax. (PDF 6725 kb) [file 12915_2019_651_MOESM4_ESM.pdf]

Figure S5

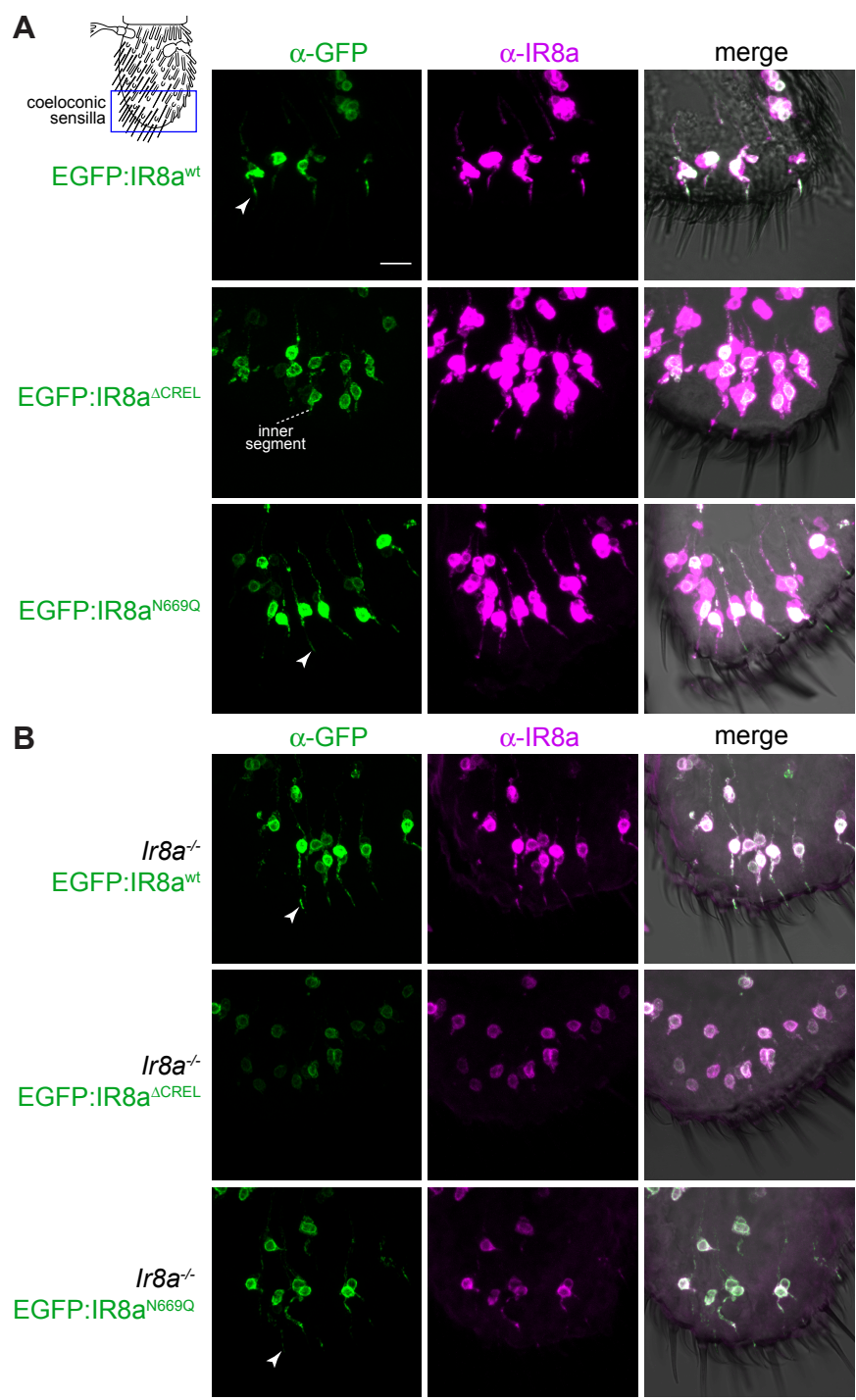

Supplement: Supplementary file 5 — Figure S5. Heterogeneous localisation properties of IR8aN669Q in coeloconic sensilla. (A) Immunofluorescence with antibodies against GFP (green) and IR8a (magenta) on antennal sections of animals expressing the indicated transgenes in Ir8a neurons. Genotypes are of the form: Ir8a-Gal4/UAS-EGFP:Ir8ax. Arrowheads mark examples of sensilla in which receptors are detected in the OSN cilia; this was determined by overlaying the fluorescence signal onto a bright-field channel, as shown in the merged images. EGFP:IR8a∆CREL does not traffic beyond the inner segment. Scale bar (for all panels in this figure): 10 μm. For each genotype, the phenotype was assessed in multiple sections of antennae from at least 20 animals from two independent genetic crosses. (B) Immunofluorescence with antibodies against GFP (green) and IR8a (magenta) on antennal sections of animals expressing the indicated transgenes in Ir8a neurons in an Ir8a mutant background. Genotypes are of the form: Ir8a1/Y;Ir8a-Gal4/UAS-EGFP:Ir8ax. Arrowheads mark examples of sensilla in which receptors are detected in the OSN cilia. For each genotype, the phenotype was assessed in multiple sections of antennae from at least 20 animals from two independent genetic crosses. (PDF 8558 kb) [file 12915_2019_651_MOESM5_ESM.pdf]

Figure S6

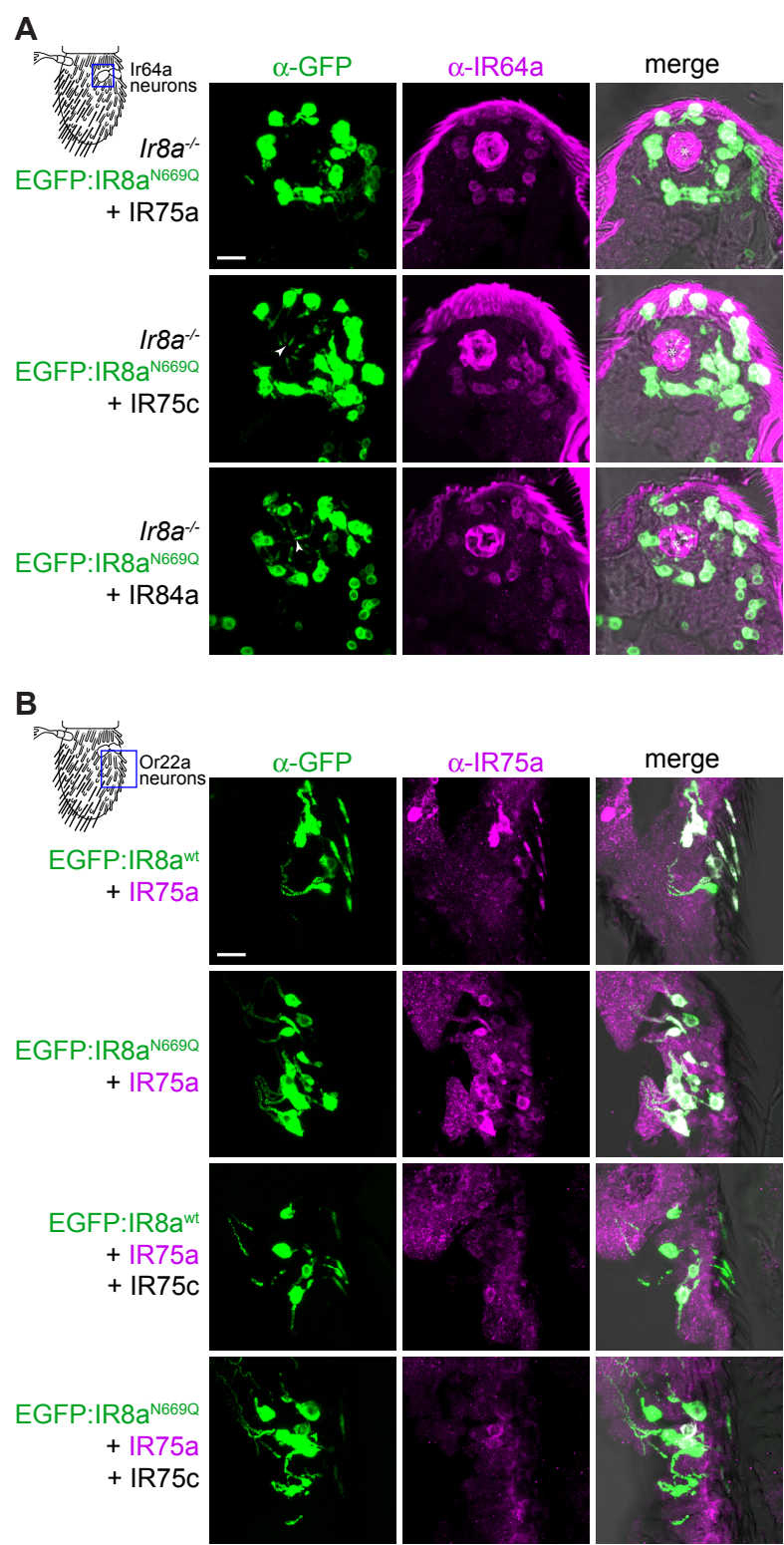

Supplement: Supplementary file 6 — Figure S6. Tuning IRs compete for, rather than assemble together with, IR8aN669Q. (A) Immunofluorescence with antibodies against GFP (green) and IR64a (magenta) on antennal sections of animals expressing the indicated transgenes in Ir8a neurons in an Ir8a mutant background. Genotypes are of the form: Ir8a1/Y;UAS-EGFP:Ir8aN669Q/UAS-IrXX;Ir8a-Gal4/+. The white asterisks in the right-hand panels indicate the central cavity of sacculus chamber 3 into which the OSN ciliated dendrites project. Due to the weak expression of IR64a in these tissues (compared to, for example, Fig. 2a), the gain setting during imaging was increased, resulting in high cuticular autofluorescence in the magenta channel, which reveals both the antennal surface and the lining of the sacculus. The arrowheads in the left-hand panels mark the ciliated endings of neurons containing EGFP:IR8aN669Q (but not IR64a). Scale bar (for all panels in this figure): 10 μm. For each genotype, the phenotype was assessed in multiple sections of antennae from at least 20 animals from two independent genetic crosses. (B) Immunofluorescence with antibodies against GFP (green) and IR75a (magenta) on antennal sections of animals expressing the indicated transgenes in Or22a neurons. Genotypes are of the form: UAS-EGFP:Ir8ax/+;Or22a-Gal4/UAS-Ir75a (top two rows) and UAS-EGFP:Ir8ax/UAS-Ir75c;Or22a-Gal4/UAS-Ir75a (bottom two rows). For each genotype, the phenotype was assessed in multiple sections of antennae from at least 20 animals from two independent genetic crosses. (PDF 12021 kb) [file 12915_2019_651_MOESM6_ESM.pdf]

Figure S7

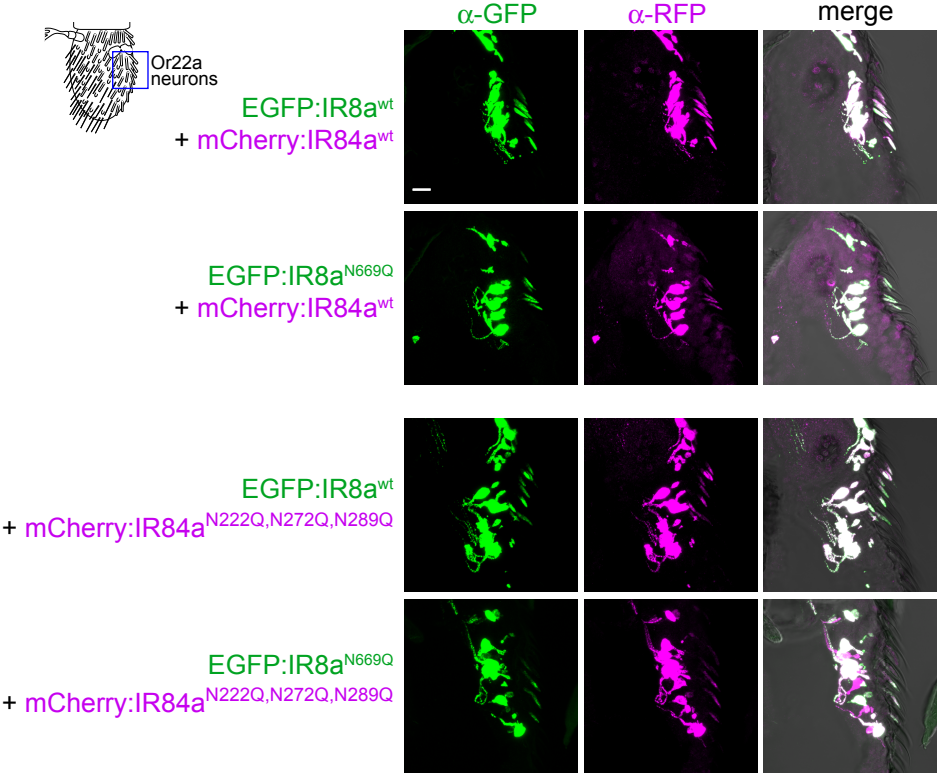

Supplement: Supplementary file 7 — Figure S7. Predicted IR84a LBD N-glycosylation sites are not essential for cilia localisation of IR complexes in the presence or absence of IR8a CREL N-glycosylation. Immunofluorescence with antibodies against GFP (green) and RFP (magenta) on antennal sections of animals expressing the indicated transgenes in Or22a neurons. Genotypes are of the form: UAS-EGFP:Ir8ax/+;Or22a-Gal4/UAS-mCherry:Ir84ax. Scale bar (for all panels in this figure): 10 μm. For each genotype, the phenotype was assessed in multiple sections of antennae from at least 30 animals from three independent genetic crosses. (PDF 4768 kb) [file 12915_2019_651_MOESM7_ESM.pdf]

Figure S8

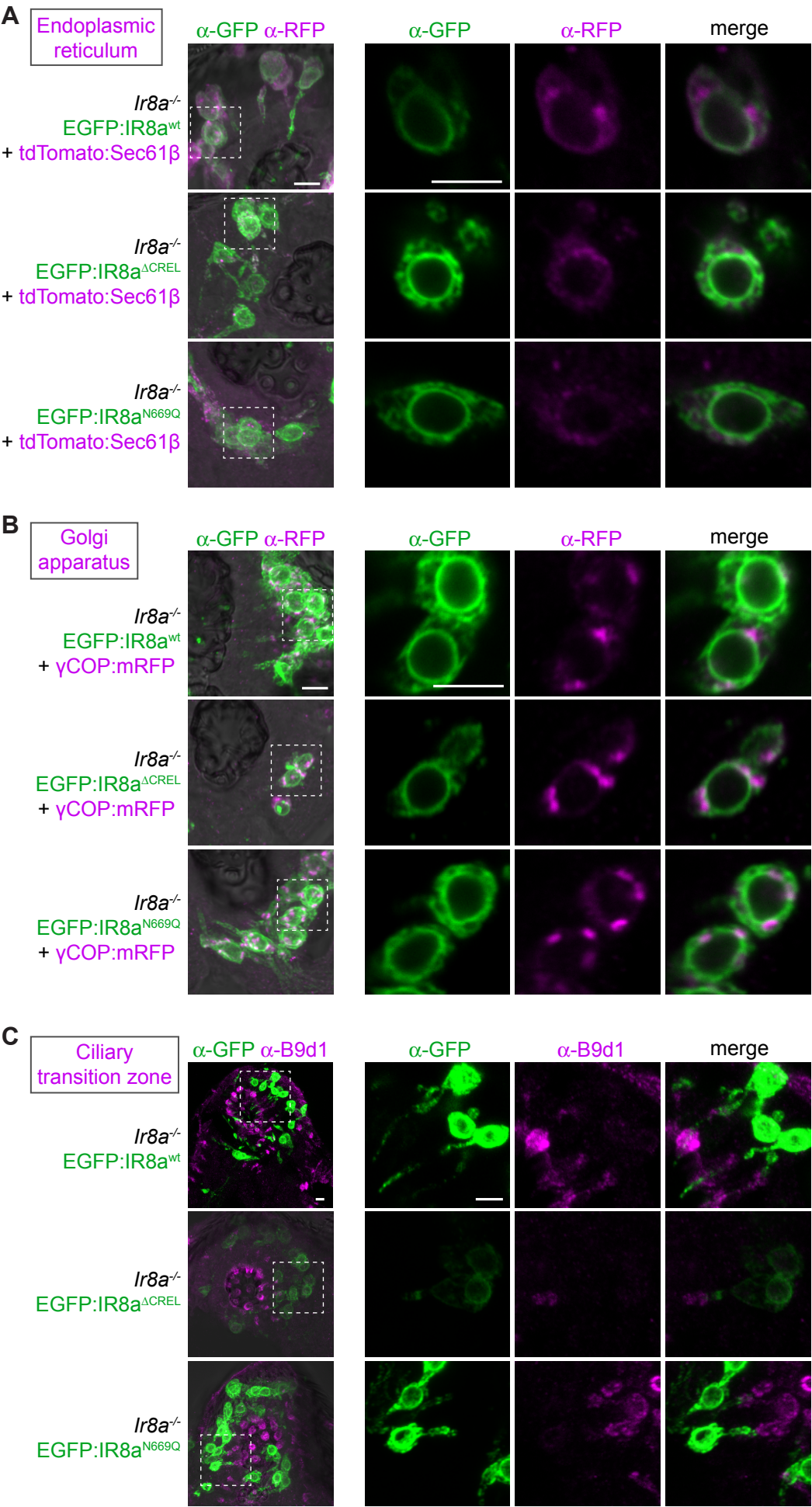

Supplement: Supplementary file 8 — Figure S8. The IR8a CREL and the CREL N-glycosylation site are important for ER export. (A) Immunofluorescence with antibodies against GFP (green) and RFP/Tomato (magenta) on antennal sections of animals expressing the indicated transgenes in Ir8a sacculus neurons. The images on the right are high-magnification, single optical slices taken within the region shown in the lower-magnification view on the left, in this and the following panels. Genotypes are of the form: Ir8a1/Y;Ir8a-Gal4/UAS-EGFP:Ir8ax;UAS-tdTomato:Sec61β/+. Scale bars: 5 μm. For each genotype, in this and the following panels, the phenotype was assessed in multiple sections of antennae from at least 20 animals from two independent genetic crosses. (B) Immunofluorescence with antibodies against GFP (green) and RFP (magenta) on antennal sections of animals expressing the indicated transgenes in Ir8a neurons. Genotypes are of the form: Ir8a1/Y;Ir8a-Gal4/UAS-EGFP:Ir8ax;UAS-γCOP:mRFP/+. Scale bars: 5 μm. (C) Immunofluorescence with antibodies against GFP (green) and B9d1 (magenta) on antennal sections of animals expressing the indicated transgenes in Ir8a neurons. Genotypes are of the form: Ir8a1/Y;Ir8a-Gal4/UAS-EGFP:Ir8ax. Scale bars: 5 μm. (PDF 9867 kb) [file 12915_2019_651_MOESM8_ESM.pdf]
